# Access to Eye Care Before and After Vision Loss: A Qualitative Study Investigating Eye Care Among Persons Who Have Become Blind

**Published:** 2020-06

**Authors:** Tosha Zaback, Stephanie Lam, Joan Randall, Teresa Field, Mitchell V. Brinks

**Affiliations:** OHSU Casey Eye Institute, Portland, Oregon, USA; Oregon Commission for the Blind, Portland, Oregon, USA; OHSU Casey Eye Institute, Portland, Oregon, USA

**Keywords:** Blindness, Access to Care, Patient Perspective, Qualitative Research

## Abstract

Navigating access to eye care requires that patients recognize the need for screening and care, employ limited financial and social resources, manage complex health insurance policies, and access specialty clinical care. We investigated the experience of patients through the progression of vision loss to blindness, utilizing qualitative methods. We conducted structured telephone interviews with 28 persons with blindness throughout Oregon. Utilizing closed and open-ended questions, we explored patient experience on the events preceding avoidable blindness. Coding for emergent themes was conducted independently by two researchers using a constant comparative method. Participants described important barriers to accessing eye care: at the systems level, lack of access to providers and treatment; at the community level, available social support and services; and at the individual level, readiness to act and trust in providers. These findings suggest that important barriers to accessing preventive eye care, early diagnosis and treatment, vocational rehabilitation, and social services often occur at multiple levels. Access to eye care should be prioritized in efforts to reduce preventable visual impairment.

## Introduction

Blindness and visual impairment affect 3.4 million people over the age of 40 in the U.S. and this number is expected to double by 2050 (Prevent Blindness America, 2012a; Varma et al., 2016). Prompt detection and treatment of eye disease can prevent up to half of these cases of vision loss, yet most of those with early sight threatening eye diseases are either undiagnosed or not receiving recommended care (Shaikh, Yu, & Coleman, 2014; Wittenborn & Rein, 2016; Wittenborn et at., 2013)

Accessing health care requires readiness to act, acceptance of the health problem, prioritizing care, and trust in health care providers (Glanz, Rimer, & Lewis, 2002). For those with eye disease, accessing care can be further complicated by the asymptomatic early course of major eye diseases and from widespread fear of blindness as well as the confusion caused by the exclusion of eye care from health insurance policies. A recent national report by the National Academies of Science, Engineering and Medicine noted the importance of individual and systems level access barriers to health care (National Academies of Sciences Engineering and Medicine [NAS], 2016). These influences are minimally addressed in national surveys. For example, the Behavioral Risk Factor Surveillance System [BRFSS] (Chou et al., 2012) survey does not adequately assess individual experiences accessing eye care providers and systems, as it captures only self-reported visual impairment and time since the last eye exam.

Access barriers to health care include economic, transportation, geographic, cultural, and health literacy (Baker et al., 1996; Chen, Vargas-Bustamante, Mortensen, & Ortega, 2016; Goins, Williams, Carter, Spencer, & Solovieva, 2005; Kullgren, McLaughlin, Mitra, & Armstrong, 2012; Macinko, Shi, Starfield, & Wulu, 2003; Schillinger et al., 2002; Syed, Gerber, & Sharp, 2013). Additional unique access barriers further hamper access to eye care. The frequent requirement of purchasing supplemental vision insurance to pay for eye exams poses a major barrier to accessing preventive eye care (American Academy of Ophthalmology [AAO], 2015). Patients 40–64 years old received fewer eye care visits and more self-reported near and distance vision difficulty when they lacked vision insurance coverage (Li, Xirasagar, Pumkam, Krishnaswamy, & Bennett, 2013). As well, visual impairment and blindness can preclude driving to eye care visits, and as few as 30 minutes or 30 miles of travel pose significant barriers for affected individuals (Probst, Laditka, Wang, & Johnson 2006, 2007; Syed et al., 2013).

In an effort to understand the patient experience, we conducted qualitative interviews in which study participants shared their experiences interacting with eye care systems. Specifically, we were interested in learning historical perspectives on navigating eye care through the course of vision loss and barriers and facilitators to accessing care. This study contributes to the literature by presenting the perspective of patients on reaching and maintaining access to vision health care at the individual, community, and systems level. Through the patient voice, we hope that previously undervalued opportunities to improve access to care can be addressed through the multiple levels of interaction identified. The results of our study will be shared broadly with eye care providers, the Oregon Academy of Ophthalmology, Oregon Commission for the Blind and national policy advocacy organizations such as PreventBlindness and American Public Health Association.

### Authors’ Role and Relationship to Research

All authors from the Casey Eye Institute (CEI) work in outreach aimed at the prevention of avoidable blindness. Teresa Field from the Oregon Commission for the Blind (OCB) is tasked with developing programs for persons with blindness to live full lives. Tosha Zaback is master level trained public health evaluator with over 15 years of qualitative research experience. In her previous career, she was an Ophthalmic Technician which prompted her to pursue prevention efforts in research, evaluation, and program implementation. Stephanie Lam is a graduate student studying community psychology with training in qualitative methods. She completed this project as an intern at the CEI. Joan Randall is a master level trained researcher and administrator with over 25 years’ experience in eye care. Joan conducted key informant interviews and all telephone interviews for the study. Mitchell Brinks is a Public Health Ophthalmologist who conceived of this project and directs all outreach efforts for CEI. The results of this project will be shared with those working in the field of eye care and used to help design programs that improve access to care.

## Methods

### Qualitative Inquiry

The use of qualitative methods is an effective strategy to investigate shared experiences and factors that influence health outcomes (Strauss & Corbin, 1998). We conducted structured telephone interviews using closed- and open-ended questions with Oregon residents with blindness recruited by the Oregon Commission for the Blind. Close-ended questions consisted of participant characteristics and questions which were used to preface the open-ended question that followed. For example, we asked how important eye care was compared to other parts of your body and had Likert scale answers. We chose to conduct a standardized open-ended interview due to the lack of patient perspective in broad population eye health questions (Dwyer-Lindgren, Mackenbach, van Lenthe, Flaxman, & Mokdad, 2016). Standardized qualitative inquiry allows for comparisons across multiple participants and helps to reduce bias based on assumptions based on personal experience in the field (Ulin, Robinson, & Tolley, 2005). The purpose of the research was to obtain participants’ views on events and experiences while interacting with health care resources during their course of vision loss to blindness.

### Recruitment

A convenience sample of legally blind participants was recruited through the Oregon Commission for the Blind counselors and teachers who identified adult persons with blindness due to macular degeneration, glaucoma, and diabetic retinopathy from both urban and rural locations around Oregon. Legal Blindness was defined in accordance with U.S. law, having a best corrected visual acuity in the better seeing eye of equal to or worse than 20/200 or a visual field of less than 20 degrees.Each participant was contacted by phone and asked if he or she would be willing to participate in the study. As each participant gave telephone assent, the interviewer (JR) requested their mailing address. A consent form was then mailed to each of them. An opt-in informed consent was mailed to potential participants across the entire state of Oregon. Upon receipt of the signed consent form from the study participants, each one was called again by JR and a telephone interview appointment was scheduled, based on the convenience and availability of study participants. Telephone interviews, ranging from13 to 56 minutes, were tape-recorded and transcribed with participants that mailed back the consent forms. Thematic saturation was reached at 28 participants.

### Participant Characteristics

Between May 2016 and May 2017, 28 telephone interviews were conducted with participants across the state. Detailed participant characteristics are listed in Table 1. All participants were 40 years or older, 57% were female, 89% Caucasian, 53.6% resided in a rural community, and most (71.4%) had at least some college education, and most (78.5%) had household incomes of $49,999 or less. Participants reported macular degeneration (57.1%) followed by glaucoma (14.3%), diabetic retinopathy (14.3%), and multiple diagnoses (10.7%) as the cause of blindness.

### Data Generation and Collection

The interview guide was informed by experienced qualitative research experts. Advisors from the Oregon Commission for the Blind reviewed and provided edits to ensure cultural sensitivity. We then tested among four independent living teachers across the state. Each teacher went through the questions with our researcher line by line and provided input on phrasing and order of questions. They also suggested omissions and additions. With each round of testing we incorporated changes until we were all confident that our interview guide was appropriate to administer to our participants. Demographic data were collected and reported above using close-ended questions. Open-ended questions included questions related to delay in obtaining care, the role of insurance in receiving care, perceived importance of eye care, and availability of eye care and treatments.

Due to financial constraints and to ensure that we had representation that covered the diverse state of Oregon, we conducted structured telephone interviews in lieu of in person interviews. Interviews were conducted by one researcher (JR). Each consented participant was reminded of the purpose, risks, and benefits to the study and their rights as a volunteer participant. The researcher obtained verbal consent to audio record the interview and explained that to protect the anonymity of participants, all identifying information from the transcripts would be removed and not included in any publications. Participants were informed that they did not have to answer any questions they did not feel comfortable answering and they could discontinue the interview at any time. All transcriptions were used as data when conducting our analysis.

The Institutional Review Board of Oregon Health & Science University reviewed and approved the study protocol. All participants provided informed consent for use of their de-identified data to be used for research purposes. The study followed the tenets of the Declaration of Helsinki.

### Analysis

We transcribed all interviews and uploaded into the software program NVivo (11.4.1.1064 ed.) from QSR (2015) which was used for data management in the identification of common themes. Anonymity was ensured by removing any references to names and identifying information. However, references to geographic locations were included to discern between rural and urban locations in the state. We chose to use an inductive approach to analyze data ensuring that we were able to understand the themes that arose from the perspective of the participants rather than a prescribed hypothesis (Corbin & Strauss, 1990; Glaser, Strauss, & Strutzel, 1968; Ulin et al., 2005). We used a constant comparative method (Addison, 1999; Pope & Mays, 1996) in our qualitative analysis with the same two researchers (SL and TZ). The data were coded thematically and analyzed for emerging themes (Glaser et al., 1968; Pope & Mays, 1996). Below we have listed in the step to ensure rigor when conducting our analysis.

- The first step taken by both researchers was data familiarization which was done by transcribing interviews and then reading each transcript. Through this process researchers created a list of categories and associated meanings independently of each other in relation to accessing eye care.
- After the first process, both researchers discussed initial impressions of the data and the categories each had identified which were then used to create larger categories by merging, subtracting, and modifying until agreement was reached on a preliminary list of categories.
- Both researchers independently defined the listed categories and came together to discuss and create agreed upon definitions to create a preliminary coding scheme. The coding scheme involved several phrases and definition of those phrases that helped guide the analysis. For example, “trust in providers” was defined as positive or negative expressions of trusting their medical providers.
- Then each researcher applied the preliminary coding scheme to same five transcripts, while looking for additional emerging categories. Throughout the process of coding the data, other nuanced emerging codes appeared, which followed the process of discussion as outlined above and then was applied to each transcript.
- In the next step of the analysis we utilized the agreed upon the coding scheme and each researcher coded the next five transcripts independently of each other. Researchers reconvened to review the results for discrepancies and to continue to define themes and subthemes following the same process as outlined above. For example, when defining “readiness for seeking eye care” we realized that simply defining this as making eye care a priority, we needed to take into account that some participants simply were not aware or in denial of the need for eye care, so we added subthemes to this category.
- In each round of coding, discrepancies were discussed until an agreement was made and codes were redefined for mutual understanding. The two met weekly and discussed the definitions of each theme according to the coding results and creating subthemes as they both dove deeper into the data. As changes were made to the coding scheme, we returned to previous transcripts to add or subtract additional coding as needed.
- The process outlined about was repeated until consensus was reached and duplicative coding yielded the same results for every transcript.
- Once all transcripts were coded, we reviewed data from each code and collapsed or separated into subthemes based on our results. For example, we originally had “transportation” as an independent code, but upon review we realized that each transcript with coding in “transportation” was also coded in “availability of eye care providers.”

This approach provided the results for this study. Presented in Table 2 are the three major themes that emerged from the data including: (a) readiness for seeking eye care, (b) trust in providers, and (c) access to eye care. In the results section we provide context and representative quotes from our interviews.

## Results

Using the interview data, we explored access to care before and after losing vision including the participants’ views on barriers and facilitators to eye care. We describe our resulting themes below. Identified themes revealed multiple levels to accessing care at the individual and environmental level. Table 2 presents an outline of our three major themes and sub themes for access to care and attitudes and readiness for seeking care. We describe our results in more detail below.

### Theme 1: Readiness for Seeking Eye Care

We defined “readiness for seeking eye care” as the attitudes and behaviors expressed by our participants in seeking eye care. Participants were asked to describe their reasons for accessing or delaying health care. Participants expressed their attitudes and behavior in seeking eye care resulting in three sub-themes that prevented timely diagnosis and treatment of eye disease: awareness, prioritizing, and denial even though most participants (75%) reported their eye health as being very important compared to the health of other parts of their body.

#### Awareness.

This subtheme included knowledge of recommendations for eye health exams (preventive and disease management) as well as a knowledge gap, “You don’t know what you don’t know.” We coded participants’ knowledge or lack of knowledge of recommendations for eye health exams including preventive exams and disease management as awareness. We asked participants if they knew the recommended schedule for eye care provider visits. Even though most (n=20) participants reported knowing the recommended eye visit schedule at the time of the interview, they described not being aware of preventive eye exams prior to learning of problems with their eye health, and often after permanent damage to their vision had likely already occurred (having only pursued eye care once they had symptoms). When asked if she knew the recommended visit schedule for preventive exams, this participant reported,“No. Before that I never really went [referring to before experiencing any symptoms]. I didn’t need glasses. I never had trouble seeing.”

When participants were asked “did you know that certain eye conditions, like cataract, glaucoma, diabetic retinopathy, or macular degeneration, could make you go blind?”, many participants (n=12) reported that even at the time of diagnosis they did not understand that their eye conditions, such as glaucoma, diabetic retinopathy, or macular degeneration could cause blindness in future which could impede their readiness to act on their disease.

#### Prioritizing.

We defined prioritizing as how quickly a person decides to take action on recommendation for eye care and how they decide to prioritize eye health. A few participants reported always prioritizing their eye health, describing professional regulations such as maintaining their pilot’s license that required eye testing. As described below under “availability of eye care providers,” participants make huge efforts including relocating and lengthy travel to access eye care. This participant was unable to access affordable eye care in another state, so he decided to move to Oregon where he was able to get eye care through Medicaid assistance: “‘cause I quit my job just to get my eyes [unintelligible], because my eye[s] are more important to me…And uhm, and I moved back up here [Oregon].”

However, some participants (n=5) reported that their eye health was not something they gave much thought until they started losing vision, four out of the five participants often expressing grief in this oversight. Once participants experienced the consequence of or fear of losing vision, eye health became a significant worry and time consuming priority in their life as described by these participants. “Until I lost my eyesight it was inconsequential. When I lost my eyesight it was extremely important.”

Before that I never really went. I didn’t need glasses. I never had trouble seeing… [Then] one day, and this came upon me. I just, everything was blurry. And I wondered why. And I thought, oh, I’m just tired. So I guess I’ll go upstairs. Because I was working in my flower garden. And then I though, wait a minute, this is not right. Something is wrong. Everything is so fuzzy. So I just got in the car and drove myself to the eye doctor. And then she checked them and said that’s where I had this. They were bleeding in the back. And she made me an appointment, for the next day to go to Salem to a retinologist.

#### Denial.

We defined denial as expressions of “It doesn’t happen to me” or “I don’t have anything,” or “I will get better on my own.” We included some individual descriptions as an inevitable part of aging and perceiving that everyone goes through the same thing. A few (n=4) participants reported described not seeking eye care because they did not believe that they would become blind or had control over the outcome for various reasons. Participants described ignoring symptoms and not seeking out eye care and treatment because they accepted it as an inevitable part of their aging as described by this participant.

I don’t know anything that we could have done that would have made a difference. Like I said, it seemed to digress real quickly after the cataract operation. But I don’t know if it’s related or not. But I’ve talked to others too that have had the same experience. So, they had the eyes operated on and wasn’t long before they got…Oh, it’s an affliction of age.

Some participants described ignoring their family history and assuming their outcome would be the same as family members regardless of intervention and advances in medicine. One participant was aware of treatment related to eye problem but did not seek treatment describing the reasons for not seeking care as “general stupidity-father and uncle had glaucoma, but didn’t pay attention, denial.”

### Theme 2: Trust in Providers

We asked participant if they “usually trust doctors and believe what they tell you.” Participants reported a mixture of negative and positive feelings about trust with their eye doctors, at both the institution and individual level. Half of our participants (n=14) reported a trusting relationship with their doctors. These participants reported that they trusted their doctor was doing all they could for them. “My doctors were very good about making sure that I came in every so often and went through all the tests.” “Well, I will tell you that he watched it very closely. We tried to keep the pressure, the eye pressure down. And I saw him probably, I would say once every 2 to 3 months.”

Some (n = 5) participants reported having no trust in their doctors, giving examples of care that they or their family members had received and that they thought was harmful, describing mistakes or miscommunications. This participant describes his interaction with the medical system and consequential distrust in doctors and harm that affected his general and eye health.

Or, I had one doctor here that I actually got his license stripped from him because I’ve had… Out in Indiana they gave me nitroglycerin. This doctor wanted to give me nitroglycerin. You know, all types of stuff. He gave me extra medicine that he knew that was going to mess up my kidney. That helped exacerbate my blindness. Even though I knew I was going blind, I couldn’t find help. Nobody would help me or tell me how… what I should eat and all this stuff. You know, [unintelligible] all that. So, it kind of exacerbate on it. You know.

Two participants described distrust in the Department of Veterans Affairs hospitals and clinics as a barrier to receiving the care they needed often deciding to forego care as illustrated below.

Well, some of the VA doctors, of course…Almost all the doctors there at the VA are from the health site center. And a lot of them I don’t trust at all. Just because, you know, they tell me one thing and they do another.

The remaining participants (n=9) proactively sought out doctors they trusted. The quote below describes one participant’s interaction with her eye care providers and her decision making process with the providers she trusts.

It’s the impact of their decision was so strong that…Well, I don’t mind taking risks, but I like to know the odds or what the chance… In this case it was somebody wanted to do some surgery on the eye for relief of pressure in the eye. Oh, excuse me. But the advantage was it was only reduced medication. Well, medications were relatively inexpensive at the time. And I said I’m not going to risk the chance of the slipping knife with some other injury or scar tissue in the eye. Plus, it cost more money. And I think that’s the only time where I really questioned the doctor.

### Theme 3: Access to Eye Care

This theme was defined as environmental reasons why patients access or delay getting eye care. We further defined the following subthemes of access to eye care: (a) availability of eye care providers, (b) insurance/cost, and (c) patient-medical systems interaction. Participants described the importance of navigating the health care environment in securing eye care. Environmental access was defined as geographic/nearby availability of providers, medical systems, and insurance coverage and out of pocket expenses.

#### Availability of eye care providers.

We defined this access to care subtheme as participant descriptions of geographic availability, travel mechanisms, and distance to receive care. Although most (n=22) of our participants reported they were able to access clinical eye care, some (n=11) reported traveling 30 or more miles for their visits. For those with vision loss, each additional mile can pose a substantially greater burden than for those with vision adequate for driving. Participants residing in rural areas traveled up to 300 miles to access specialty eye care and had few transportation options. Although a few communities offered public transportation services, most relied on family and friends for transportation as lengthy trips often required a full day away from home and home for patient and family support. Two participants moved to urban areas to lessen this burden on their social networks. Those without social networks able to provide transportation support had little access to low vision services in non-service areas. Even those living in metropolitan areas described long commutes to access the specialty care via public transportation. This participant undergoing treatment with specialists described multiple transportation barriers to accessing care.

I would have had to drive 300 miles, one way. Now see, we have a local doctor who does eye glasses in John Day, which is about 35 miles one way. And then she referred me to another one over in Bend [150 miles from John Day]. And then I had to drive all the way over there. My first treatment was in Portland because there were no doctors in Bend at that time. And then in the winter time we have to take the people mover [transportation services] to Bend to the doctors.

This participant describes an option that she believed could have been beneficial to her eye health, but the distance and transportation barriers prevented her from participating.“I got an invitation to one [referring to a clinical trial] in Portland. But I couldn’t go because I can’t drive. I can’t get anybody to take me there. So, I didn’t do that.” One participant residing in eastern rural Oregon reported long travel for eye care but did not voice a burden. However, this participant also described a personal support system. “Wednesday I had my pastor drive me in to Portland. Visudyne. That’s an operation on the eye.”

#### Patient - medical systems interaction.

This subtheme of access was defined as different layers to fully accessing care/medical systems/providers. It includes patients’ perceptions of navigating healthcare systems and their reports of barriers and facilitators to care. Participants expressed confusion or disappointment with the complex nature of accessing care from their primary care physician, comprehensive eye doctor, and specialty care from the intake staff scheduling appointments as well as provider as described by this participant.

And so last time I called, you need to know this. I was just exasperated because they won’t address the left eye. I’m getting older, not getting any younger. And I’m tired of not being able to see. I called and asked to speak to the doctor that removed the cataract. And do you know what they said? You can’t talk to him. I said, excuse me? He’s my doctor. No, he’s not your retina specialist. It needs to go through him. And I said, no. I need to talk to the guy that removed the cataract. She goes, well, I can’t let you do that. Twice, I tried to call. And they won’t let me talk to him. Now, that doesn’t sound right. Why don’t they want me to talk to him?

Participants expressed concern and frustration over the loss of connection to their eye doctors, who, in spite of their leading role in eye health, seemed unprepared and disengaged to navigate the patient’s course after they had become blind. Participants reported a lack of, or ineffective, communication as a barrier to preventive and low vision care, recommending that providers take more time to understand the patient’s circumstances and ensure that they understood follow-up recommendations as well as low vision resources available to them. This participant describes his experience in this process.

That was one of the biggest concerns…They basically let me out the back door. Or I walked out the back door. They didn’t guide me at all with vision options. And that’s one of my complaints. And when I talked to my comrades in the blind community, the same thing. If they can’t salvage the sight, they lose interest or lose income. That’s the cold way to do it.

#### Insurance and cost.

We defined this subtheme of access to care as coverage that is included or excluded for care or the lack of knowledge of what care is provided because of medical jargon. We also included personal resources cost: monetary, time, and/or emotional costs associated with accessing eye care. Vision and eye health insurance is often provided separately from standard insurance policies, and this additional coverage was a key influence on access to eye care. For patients 65 years and older, like those interviewed here, Medicare provides the basis for most insurance coverage, though it does not include “vision” coverage for corrective lens measurements and purchase, supplemental plans can be purchased to cover these expenses. Difficulty with these processes, for patients or clinic staff, include anticipating whether an eye exam will qualify for their insurance coverage. “Medicare pays for the glasses. But they wouldn’t pay for the refraction.”

Study participants reported difficulties accurately conveying their concerns and needs to both the insurance company and clinical staff. Although, most (n=25) participants reported some type of insurance coverage over their course of vision loss, some (n=8) still delayed getting care. Those that delayed getting care reported the cost of eye care as their biggest barrier. “Okay, then here it says oh we, we don’t pay a part, that’s part of the surgery. So, I had to come up with 300 and some dollars.”

For some living on a fixed income, coming up with 300 dollars can mean significant sacrifice. Participants also described frustration with insurance companies. Participants described feeling uncomfortable and confused by provider’s approaches to insurance billing (e.g., a seemingly inaccurate diagnosis) in order to receive payment.

[Participant describes her interaction with her doctor] “Is this caused from my lymphedema? He [Participant’s doctor] goes, if I write down lymphedema, I don’t get paid. So I’m going to say it’s caused from your diabetes. Are you on board here?”

## Discussion

Results from our study suggest that for many, the value of eye care was not recognized until they personally experienced vision loss, and at times only after a diagnosis of eye disease accompanied vision loss. The often strong sentiments of fear and value associated with blindness may trigger denial of the need for eye care, as was observed among these study participants (Scott, Bressler, Folkes, Wittenborn, & Jorkasky, 2016). These sentiments place added demands on the trust these study patients had for their eye doctors. As well, blinding eye diseases may present with few symptoms, or symptoms may be erroneously attributed to the need for corrective lenses. Thus, several sources may contribute to delayed access to clinical care. Our findings did not indicate that income significantly influenced awareness of the importance of eye care. Several investigations have documented the need to enhance public health educational campaigns aimed at changing the societal and environmental climate around valuing eye care. Programs to improve awareness would do well to recognize the need to address this issue across the full range of socioeconomic status (Alexander, Miller, Cotch, & Janiszewski, 2008; NAS, 2016; Prevent Blindness America, 2012b; United States Preventive Task Force [USPTF], 2013, 2014).

Participants reported limited prioritization in part because of the added challenges of navigating insurance and medical systems when seeking eye care. In the United States, the “add-on benefit” status of vision insurance is often a separate entity from standard health insurance. Those without these additional insurance policies usually pay the full cost of eye care directly. If they are not experiencing symptoms they often choose to prioritize other health concerns or financial obligations. Even those with some form of vision health insurance may struggle to accurately convey the reason for their visit to medical intake specialists adding confusion to the analysis of if they qualify for a medical insurance covered eye care exam, rather than a vision category insurance exam. While those working in clinical eye care may understand the differences between medical eye examinations and vision examinations, the general public and primary care practitioners often do not.

Unlike other health problems such as high blood pressure, those with visual impairment may not be able to drive and may need to rely on family, friends, or public transportation to reach eye care. Even modest vision loss, when compounded by the visual deficits induced by eye exams (e.g., pupil dilation), can limit independent travel to eye clinic visits. As sight threatening eye disease often requires specialty clinical care, some patients, especially those living in rural areas remote from eye care specialists, may need to travel significant distances. The greater travel demand further increases dependency on social support networks for rural patients and further highlights the importance of transportation services to maintain access to care.

The patient perspective offers important information which can help improve eye care. The patients studied here have extensive experience, having navigated eye care systems for many years through the course of gradual loss of vision caused by eye disease. The findings from this investigation suggest that barriers to accessing preventive eye care, treatment, and low vision support occur throughout varied aspects of their efforts to access eye care. Key patient perspectives were newly uncovered informing data gaps and concerns raised throughout the literature and highlighted in the NAS, (2016) “Making eye health a population health imperative: Vision for tomorrow” report which encouraged research on patient perspective and the following strategies: (a) designing public health campaigns, (b) improving access to eye care through insurance policy revisions, (c) decreasing the travel burden to eye care providers, and (d) improving programming for those with visual impairment. Our results provide the patient perspective specific to eye care access that the NAS, (2016) calls for and highlights the specific access issues for eye care which are similar to healthcare as a whole but also unique. Unlike other preventive health exams for early detection and treatment of disease such as breast, colorectal, or cervical cancer, accessing eye care requires an awareness of the importance of preventive eye exams that is often unknown or not prioritized due to the asymptomatic progression of eye disease. Furthermore, accessing eye care requires a sophisticated understanding of what is covered or not covered by health insurance or vision insurance. In contrast, general healthcare does not require this level of understanding and navigation of complexities that require using the correct terminology just to get an appointment at the eye doctors’ office.

## Limitations

As a convenience sample, the data may not be inclusive of Oregon’s varying demographics and social settings, as most participants were white, insured, and not all regions of the state are equally represented. Participants were recruited not only by informational material mailed to OCB registrants with macular degeneration, glaucoma, and diabetic retinopathy, but also by OCB counselors who could have introduced bias. However, we think that utilizing counselors’ expertise in participant selection was a strength of the study providing us with participants with vast experience in navigating the loss of vision through the eye care system which is similar throughout the United States. Most (see Table 1) participants were white, insured, experienced macular degeneration as the cause of blindness, 21% of the population had a college degree, and not all regions of the state are equally represented. However, this compares well to the population of Oregon (87% White, 25% Bachelor degree) and characteristics of the Oregon Commission for the Blind Registry (White 85% and macular degeneration 35%) (Brinks et al., 2019). Despite efforts to include an equal number of participants experiencing macular degeneration, glaucoma, and diabetic retinopathy, participation was predominantly from those with macular degeneration (57.1%), glaucoma (14.3%) and diabetic retinopathy (14.3%). As macular degeneration tends to cause blindness later in life and is especially common among Whites, our sample may be biased away from younger and more racially and ethnically diverse populations. Nuanced perspectives from underrepresented groups may be different from the findings reported here. Other populations may have different experiences accessing eye care, perhaps those from trust (or lack of) or integration into the health care system. The sometimes historically remote course of vision loss, interview questions, age, and mood at the time of the interview may have altered participant responses as well (Corwin, Krober, & Roth, 1971; MacQueen, Galway, Hay, Young, & Joffe, 2002; Schacter, 1987).

### Implications of Research and Practice

In the U.S., vision loss is often preventable if clinical care can be accessed in a timely manner. Additionally, earlier access to vocational rehabilitation programs significantly improve the quality of life after vision loss has occurred. Multiple access barriers to eye care and vision rehabilitation prevent much of the potential impact from the advanced eye care system in the U.S. Adding the perspective of patients navigating the eye care system addresses an important knowledge gap for efforts to improve eye health in the U.S.

Our findings suggest that patients primarily rely on their eye doctors to navigate systems of care. Doctor-patient communication gaps identified here raise significant concerns about their potential damaging effects on adherence to recommended treatment and future exams. Despite clinical advances, patients who leave clinic visits unaware of the details or implications of their eye disease are less likely to adhere with care recommendations. Eye doctors were also perceived as sometimes unprepared to guide patients to low vision rehabilitation services. This experience prompted disappointment among participants who retained the view of their eye doctors as the key caretaker of their visual health, including after the onset of legal blindness. Health care providers may be unaware or uncomfortable recruiting social services and resources outside of their familiar clinical setting to guide their patients through the process of accessing low vision care. Social services are particularly important to support access to eye care and visual rehabilitation programs as vision loss occurs. With the right support and resources, our participants demonstrated resiliency and high quality of life. Bridging the gap between medical eye care, low vision support, and rehabilitation is imperative to addressing key gaps in this process of care, particularly in rural areas. Investments in professional educational programs to better prepare eye care providers to address these care transitions with skill and compassion could improve the patient experience tremendously. The findings of our study suggest that accessing preventive eye care, early diagnosis and treatment, and low vision support can be complicated and barriers can occur at the policy, medical and provider system, and at the individual levels of interacting with systems of care and social services. We need to address access to eye care through public health and social service interventions, system improvements in the medical community, and through modifications to insurance and allowable coverage. Future studies should focus on the evaluation of preventive strategies to resolve key barriers and improve early diagnosis and treatment. In addition, comparison studies with other states, specifically investigating the variance in insurance coverage, geographic, and racial/ethnic barriers unique to particular states, will offer further insight into how to design effective strategies to access eye care and services.

## Figures and Tables

**Table 1:** 

*Participant Characteristics (n=28)*	n (%)
Age	
40–59	5 (17.9)
60–69	6 (21.4)
70–79	7 (25.0)
80+	10 (35.7)
Gender	
Female	16 (57.1)
Male	12 (42.9)
Race	
American Indian or Alaska Native	1 (3.6)
Black or African American	1 (3.6)
Native Hawaiian or other Pacific Islander	1 (3.6)
White	25 (89.3)
Education Level	
Completed some high school	4 (14.3)
Completed high school	4 (14.3)
Completed some college	11 (39.3)
Graduated from college	6 (21.4)
Graduate degree	3 (10.7)
Household income at onset of vision loss	
Less than $20,000	9 (32.1)
$20,000 to $34,999	7 (25.0)
$35,000 to $49,999	6 (21.4)
$50,000 to $74,999	2 (7.1)
$75,000 to $99,999	2 (7.1)
$100,000 to $149,999	1 (3.6)
$150,000 or more	1 (3.6)
Urban or Rural	
Rural	15 (53.6)
Urban	13 (46.4)
Diagnosis	
Macular Degeneration	16 (57.1)
Glaucoma	4 (14.3)
Diabetic Retinopathy	4 (14.3)
Multiple diagnoses	3 (10.7)
Other	1 (3.6)

**Table 2.** Coding themes

Main theme	Sub-theme
**Readiness for seeking eye care**	
	Awareness Prioritizing Denial
**Trust in providers**	
**Access to care**	Availability of eye care providers Patient- Medical systems interaction Insurance/Cost
